# Novel Single-Stranded DNA Circular Viruses in Pericardial Fluid of Patient with Recurrent Pericarditis

**DOI:** 10.3201/eid2210.160052

**Published:** 2016-10

**Authors:** Sébastien Halary, Raja Duraisamy, Laura Fancello, Sonia Monteil-Bouchard, Priscilla Jardot, Philippe Biagini, Frédérique Gouriet, Didier Raoult, Christelle Desnues

**Affiliations:** Institut Hospitalo-Universitaire Méditerranée-Infection, Marseille, France (S. Halary, R. Duraisamy, L. Fancello, S. Monteil-Bouchard, P. Jardot, P. Biagini, F. Gouriet, D. Raoult, C. Desnues);; Aix-Marseille Université, Marseille (S. Halary, R. Duraisamy, L. Fancello, S. Monteil-Bouchard, P. Jardot, P. Biagini, F. Gouriet, D. Raoult, C. Desnues);; Unité de Recherche sur les Maladies Infectieuses et Tropicales Emergentes, Marseille (S. Halary, R. Duraisamy, L. Fancello, S. Monteil-Bouchard, P. Jardot, F. Gouriet, D. Raoult, C. Desnues);; KU Leuven, Leuven, Belgium (L. Fancello);; Unité Mixte de Recherche, Marseille (P. Biagini);; Etablissement Français du Sang, Marseille (P. Biagini);; Centre National de la Recherche Scientifique, Marseille (P. Biagini, C. Desnues)

**Keywords:** pericarditis, pericardial fluid, ssDNA circular viruses, single-stranded DNA circular viruses, CRESS-DNA virus, gemycircularvirus, metagenomics, viruses

**To the Editor:** Circular replication initiation protein (Rep)–encoding single-stranded DNA (ssDNA) (CRESS-DNA) genomes are found in diverse group II virus families, which all possess a conserved Rep-encoding gene and a nonenveloped icosahedral capsid, except geminiviruses, which have twinned particles (*1*). Gemycircularvirus (GcV) were initially discovered in fungi, but a growing number of new species has been characterized by metagenomics in air, sewage, insects, and feces from a broad range of vertebrates (*1*–*5*). GcVs have also been found in the brain and serum of humans with multiple sclerosis; in the cerebrospinal fluid of a patient with encephalitis; and in several blood samples, including those from an HIV-positive blood donor (*6*–*8*). We report the presence of 2 divergent GcVs and a novel CRESS-DNA virus (CV) in 2 pericardial fluid samples from a patient with idiopathic recurrent pericarditis.

The patient, a 14-year-old girl who had thoracic scoliosis surgery in 2007, was admitted to the hospital in 2009 for pleuropneumonia and pericarditis, which required pericardial drainage twice within 3 weeks (samples PF_1_ and PF_2_, respectively). She had thrombocytopenia, a leukocyte count within the normal range, and a high C-reactive protein level. Biochemical and cytologic testing, bacterial cultures, and PCR of pericardial fluid samples for cytomegalovirus, varicella zoster and herpes simplex viruses, parvovirus B19, fungal 18S rRNA, bacterial 16S rRNA, and *Mycobacterium tuberculosis* were negative. Upon approval from the Institut Fédératif de Recherche IFR48 Ethics Committee (Marseille, France) and written informed consent from the patient’s parents, we submitted the drainage samples for further investigation.

Virus particles in 0.45-μm filtrates were purified and analyzed by metagenomics as described (*9*); resulting contigs were aligned against the National Center for Biotechnology Information nonredundant protein database using blastx (https://blast.ncbi.nlm.nih.gov/Blast.cgi). Three contigs were of viral origin (viral first hit, E-value ≤1E-03), all belonging to the ssDNA circular viruses. We obtained complete genomic sequences by PCR with ad hoc primer pairs and Sanger sequencing technology (Technical Appendix Table).

We annotated genomes using GeneMark (heuristic parameters; http://exon.gatech.edu/GeneMark/) and EMBOSS palindrome (http://emboss.bioinformatics.nl/cgi-bin/emboss/palindrome). Analysis of PF_1_ enabled characterization of HV-GcV1 (GenBank accession no. KU343136) (Figure). This genome displayed a typical GcV architecture, with a 2,264-nt circular DNA molecule carrying a capsid gene on 1 strand and 2 genes on the opposite strand, which coded for Rep1 (involved in replication initiation) and Rep2 (involved in replication termination), respectively. A putative hairpin structure showed a nanonucleotide motif, which was thought to be the Rep target, TAATGTTAT. A fourth gene with no homologs in databases was predicted upstream of the capsid gene. Phylogenetic inference from concatenated Rep placed this virus close to another GcV (found in sewage) in a clade comprising 2 other human-associated GcVs (Technical Appendix Figure 1).

**Figure:**
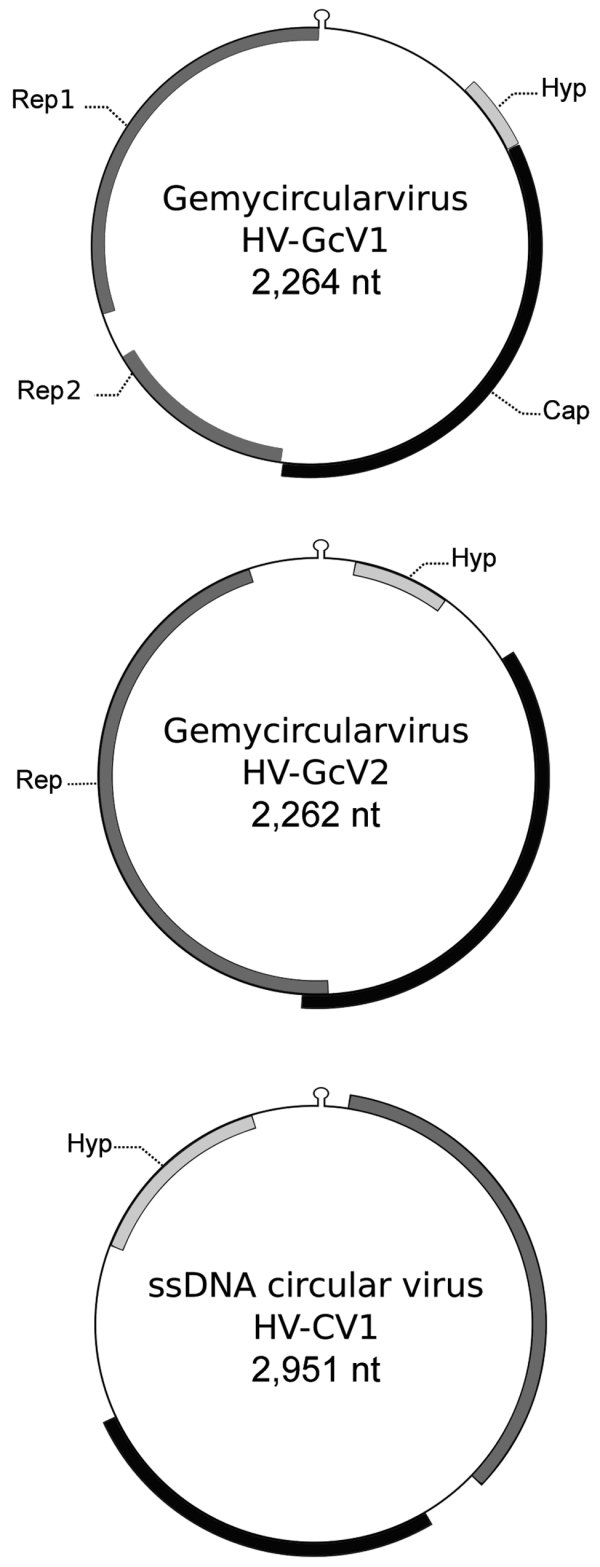
Genomic features of gemycircularviruses HV-GcV1 and HV-GcV2 and of a novel circular single-stranded DNA (ssDNA) virus, HV-CV1, including hairpin structure and predicted open reading frames. Cap, capsid; Hyp, hypothetical protein with unknown function; Rep, replication initiation protein.

PF_2_ contained 2 other viruses: HV-GcV2 (GenBank accession no. KU343137), another GcV, and HV-CV1 (GenBank accession no. KU343138), a novel CRESS-DNA virus. HV-GcV2 (2,262 nt) shares the same stem-loop motif and genomic structure with HV-GcV1, with the exception of the *rep* gene, which is coded by a single open reading frame. HV-GcV2 proteins share low sequence similarity with HV-GcV1 proteins (33% for capsid and 46% for Rep, as determined by blastp [https://blast.ncbi.nlm.nih.gov/Blast.cgi]). HV-GcV2 belongs to another clade of the phylogenetic tree that also contains sewage- and bird feces–associated viruses (Technical Appendix Figure 1). HV-CV1 (2,951 nt) possesses characteristics of CRESS-DNA genomes. Phylogenetic analysis of REP sequences showed that HV-CV1 and its closest homologue, an ssDNA circular virus of unknown taxon discovered in an Antarctic shelf pond, are distantly related to other CRESS-DNA viruses (Technical Appendix Figure 2). HV-GcV2 and HV-CV1 displayed no capsid protein similarity between them or with any other virus, as determined by blastp. Annotation of the HV-CV1 capsid gene required use of HHBlits (https://toolkit.tuebingen.mpg.de/hhblits), a more sensitive algorithm (E-value = 1.2^E-06^, probability of 97.2%).

PCR confirmed the absence of HV-GcV1 in PF_2_ and HV-GcV2 and HV-CV1 in PF_1_, suggesting multiple infections before each pericarditis event or a rapid fluctuation in the load of all 3 persisting viruses. An additional blastx search on 53 other virus metagenomes sequenced from pericardial fluids after pericarditis events failed to retrieve these sequences. To exclude the possibility of sample contamination during procedures, we simultaneously treated a sample with the same reagents and kits used for PF_1_ and PF_2_ and surveyed it by PCR; results were negative. All metagenomes are publically available in the METAVIR (http://metavir-meb.univ-bpclermont.fr) directory under the pericardial fluids heading.

No relationship between these viruses and pericarditis was established. However, the fact that some CRESS-DNA viruses are animal pathogens (*10*) and the growing number of GcVs found in human samples in pathologic contexts (*6*,*7*) indicate that the viral genomes described here might replicate in human cells, possibly as opportunistic pathogens (*8*). On the other hand, although diagnostic tests ruled out fungal or bacterial infections, we should still consider the possibility that these viruses infect other uncharacterized organisms. The genomes described here will assist further studies of the prevalence of these viruses in human populations.

Technical Appendix Maximum likelihood phylogenetic inference of gemycircularvirus replication initiation proteins (REPs) and of closest homologues of circular single-stranded DNA virus HV-CV1 REP, and PCR primer pairs used to recover whole-genome sequences.
