# Supplementary material for: Novel Single-Stranded DNA Circular Viruses in Pericardial Fluid of Patient with Recurrent Pericarditis
Source: Emerg Infect Dis. 2016 Oct;22(10):1839–41. doi: 10.3201/eid2210.160052 (PMC5038422; doi:10.3201/eid2210.160052)
Supplement: Technical Appendix — Maximum likelihood phylogenetic inference of gemycircularvirus replication initiation proteins (REPs) and of closest homologues of circular single-stranded DNA virus HV-CV1 REP, and PCR primer pairs used to recover whole-genome sequences. [file 16-0052-Techapp-s1.pdf]

Article DOI: http://dx.doi.org/10.3201/eid2210.160052

Novel Single-Stranded DNA Circular Viruses in Pericardial Fluid of Patient with Recurrent Pericarditis

Technical Appendix

Technical Appendix Table. PCR primer pairs used to recover whole-genome sequences of gemycircularviruses

Primer pair	Forward primer sequence 5′→3′	Reverse primer sequence 5′→3′
HV-GcV1–1	TTATATGCCCAGACGGACCC	ATTGTGCGGCGGATAGGATA
HV-GcV1–2	CGAATTTAACCCCGGATGCA	AAGGATGCCACCCGAATGTA
HV-GcV1–3	TTGTTCGATCAGACCACCGA	GTTCCTTCCGAGCTACAAGT
HV-GcV1–4	TCGATGTTAACTCCCTCCGG	GAAACGTGTAGATCGGCGAC
HV-GcV2–1	TTGCTCTCAACCGAAAACGG	GCCTGTTACATCGCCGATTT
HV-GcV2–2	TCCGCGTCTTTTGTAGGTCT	AAAGCGTGTGATACCCAAGC
HV-GcV2–3	TCCTTCACCAGCCTCAAGTG	TCTCCGCATGATCAGATCCG
HV-CV1–1	GGAGTTGCATGGGATCGAAC	ACCTTGAGTGACATGAGCCA
HV-CV1–2	TGGGAGTTTGGGGAGATGTC	GAATGTAGTTGGTGCAGGGC
HV-CV1–3	ACGGGCTACACATTCAGCAT	TAGACGCAGAGGATGGATCG
HV-CV1–4	AAGCTTCTTCTCGAACGTCTT	GTTGAAGGACTGGCGGAATC

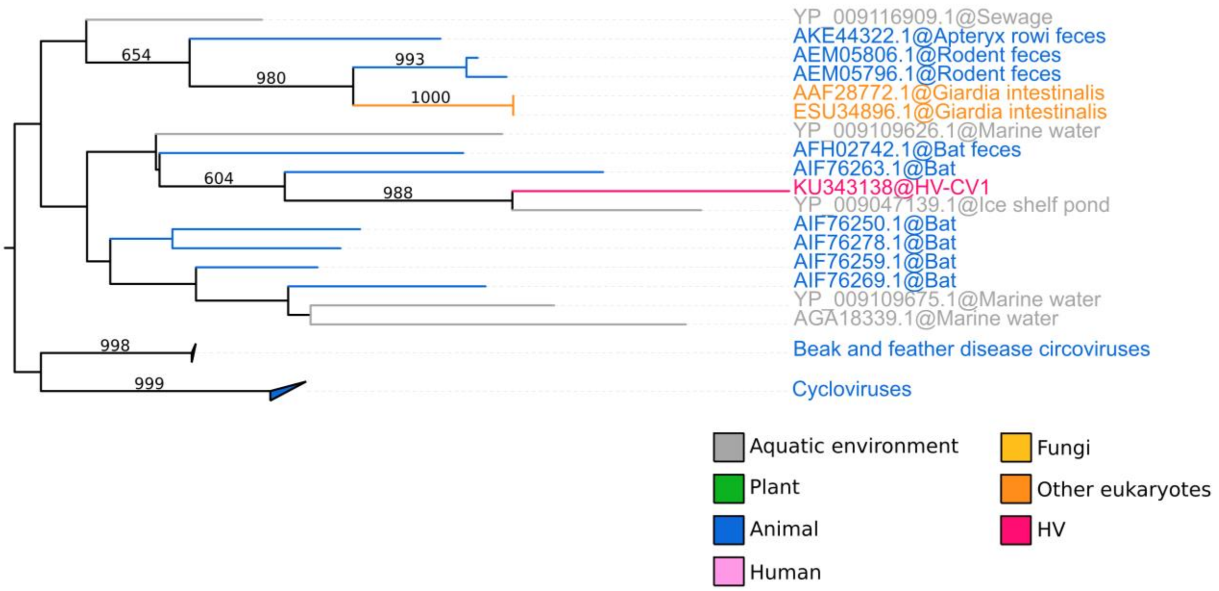

Technical Appendix Figure 1. Maximum likelihood phylogenetic inference of gemycircularvirus replication initiation proteins. Numbers on branches correspond to bootstrap values deduced from 1,000 replicates. Only scores >600 are displayed.

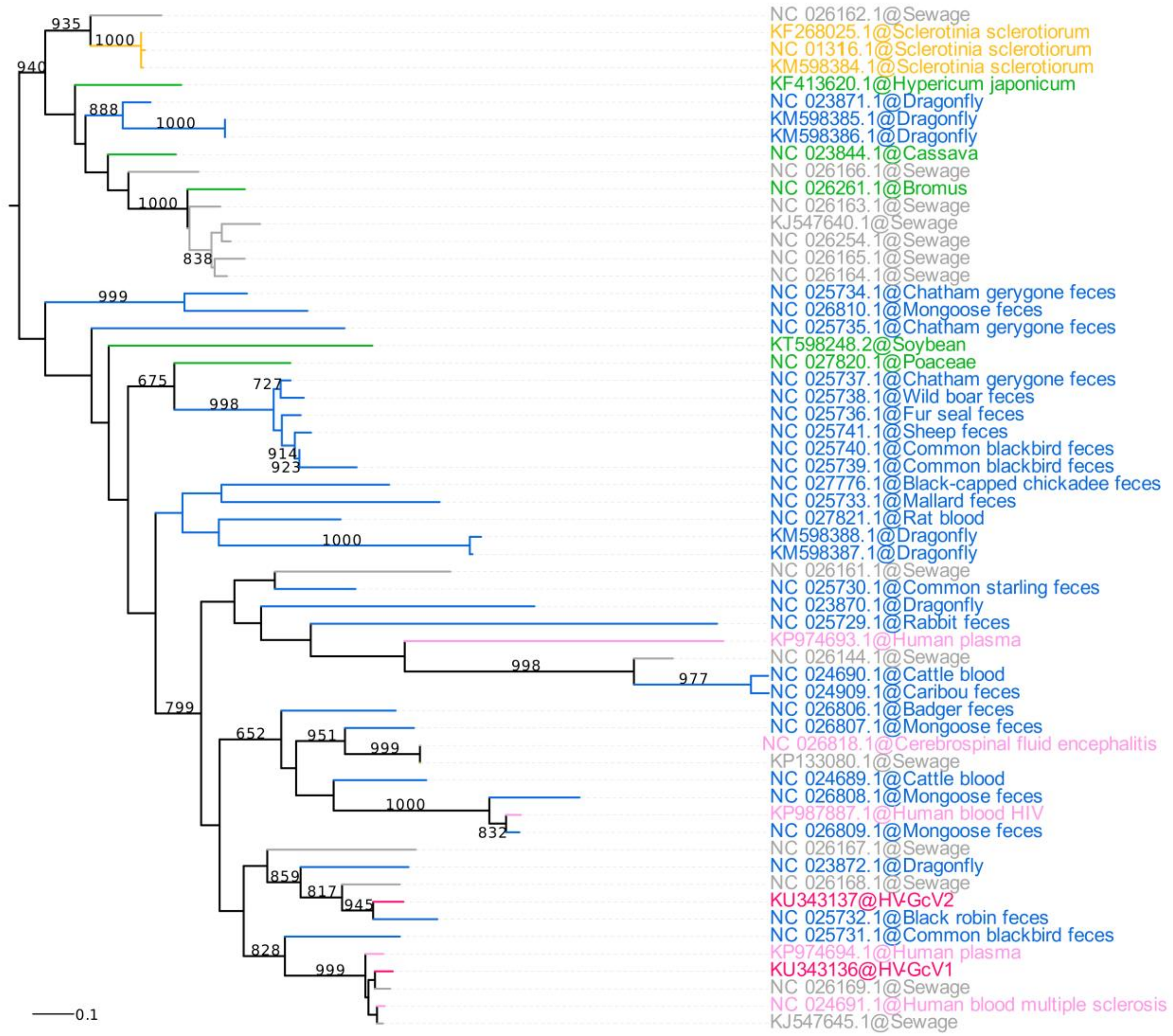

Technical Appendix Figure 2. Maximum likelihood phylogenetic inference of the closest homologues of the HV-CV1 replication initiation protein. Numbers on branches correspond to bootstrap values deduced from 1,000 replicates. Only scores >600 are displayed. Scale bar represents 0.1 residue substitution per site.
